# Dose-dependent effects of curcumin on 22Rv1 prostate cancer cell line

**DOI:** 10.1007/s11033-025-10448-9

**Published:** 2025-03-26

**Authors:** Giovanni Tossetta, Sonia Fantone, Elena Marinelli Busilacchi, Daniela Marzioni, Roberta Mazzucchelli

**Affiliations:** 1 Department of Experimental and Clinical Medicine, Università Politecnica delle Marche, 60126 Ancona, Italy; 2 Scientific Direction, IRCCS INRCA, 60124 Ancona, Italy; 3 Hematology Laboratory, Department of Clinical and Molecular Sciences, DISCLIMO, Università Politecnica delle Marche, 60126 Ancona, Italy; 4 IRCCS INRCA, 60124 Ancona, Italy; 5 Department of Biomedical Sciences and Public Health, Section of Pathological Anatomy, Università Politecnica delle Marche, 60126 Ancona, Italy

**Keywords:** Curcumin, Prostate cancer, Natural compound, 22Rv1, Cell cycle, Proliferation

## Abstract

**Background:**

Prostate cancer (PCa) is the second most frequent cancer type in the male population over 66 years. Curcumin is a polyphenolic natural compound extract from the rhizomes of *Curcuma longa* Linn (*Zingiberaceae* family) which showed important anticancer effects by inhibiting cell proliferation and inducing apoptosis in several cancer types. Recently, some studies reported that oral curcumin lowered PSA levels, but it did not modify the clinical outcomes in patients with prostate cancer who received intermittent androgen deprivation (IAD). Other studies reported that high concentrations of curcumin were toxic for patients.

**Methods and results:**

In this study we showed that low doses of curcumin can induce senescence-like effects in 22Rv1 cell line while higher concentrations have cytotoxic effects. Five,15 and 30 µM curcumin blocked cell cycle in G2/M phase but only 15 and 30 µM curcumin induced cell death. In addition, an increased expression of p21, a known senescence marker, was detected in 22Rv1 cells treated with curcumin in every experimental condition. However, the expression of p16, another known senescence marker, increased only to 30 µM curcumin.

**Conclusion:**

In the context of personalized approach in PCa care, we suggest that the appropriate concentration of curcumin used in combination with radiotherapy or with androgen deprivation therapy (ADT) could be taken into consideration.

## Introduction

Prostate cancer (PCa) is the second most frequent cancer, after lung cancer, affecting men and is the fifth leading cause of cancer-related death [1, 2]. Although prostate cancer is a biologically heterogeneous disease, most cases are localized and are highly curable by surgery, radiotherapy or focal therapy [3–5]. However, in case of hormone-sensitive metastatic prostate cancer, patients are treated with androgen deprivation therapy (ADT) even combined with second generation of anti-androgens or with trimodal therapy in select patient groups [6–8]. ADT response is very variable, and several patients become refractory to treatment and will progress to lethal castration-resistant prostate cancer (CRPC) [9–12].

Nowadays life expectancy is higher than in the past decades and it is accompanied with an increasing number of older patients with cancer. Cancer incidence is higher in older compared to younger people principally due to increased oxidative stress and DNA damage, elevated number of senescent cells and a progressive decay of immune function [13].

The majority of PCa are diagnosed in older men (median age 66 years), with a 20% in men older than 75 years due to comorbidities in older patients, PCa can exacerbate the patient’s already compromised health status promoting adverse events and drug–drug interactions [14, 15]. ADT is the leading therapy used in PCa but has important side effects regardless of the time of administration, in fact, ADT has been associated to sarcopenia and cardiovascular events. Moreover, most of the elderly have preexisting cardiovascular pathology and ADT could increase the risk of cardiovascular disease-dependent death [16].

**Fig. 1:**
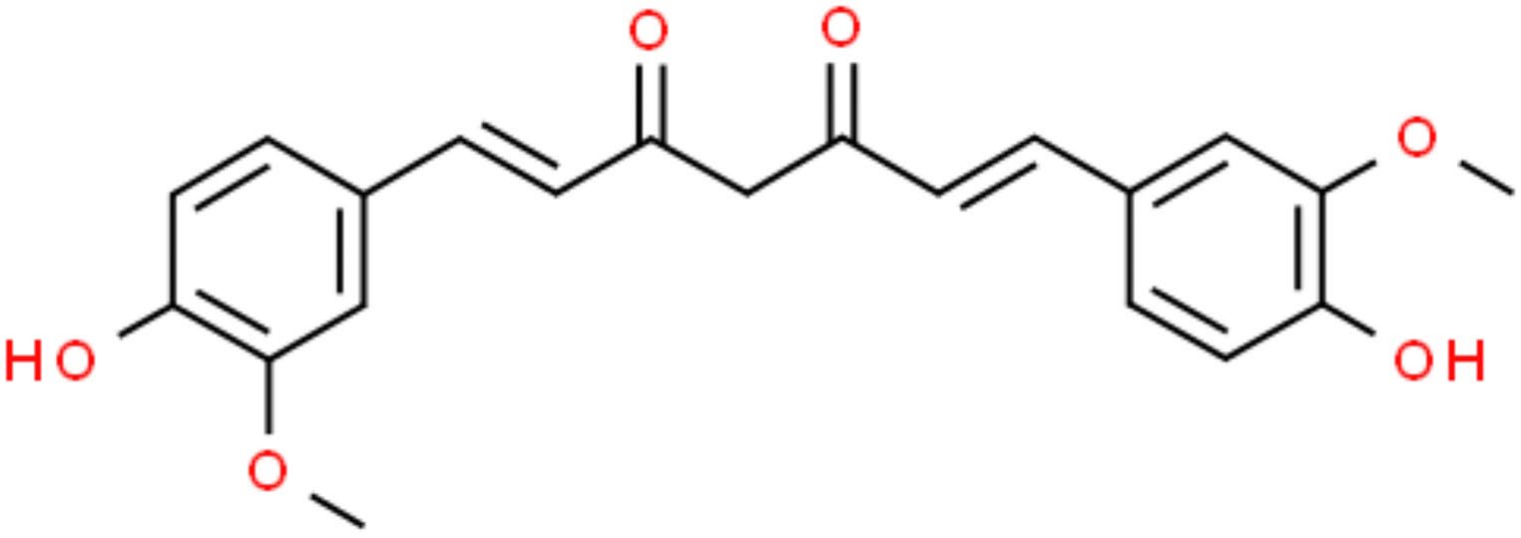
Curcumin chemical structure. The chemical structure has been taken from ChemSpider free database (https://www.chemspider.com)

Curcumin (1,7-bis(4-hydroxy-3-methoxyphenyl)-1,6-heptadiene-3,5-dione) (Fig. 1) is a polyphenolic natural compound extract from the rhizomes of *Curcuma longa* Linn (*Zingiberaceae* family) [17, 18]. Since in humans curcumin did not show any toxic effect at the dose of 6 g/day orally for 4–7 weeks, this natural compound is generally recognized as safe for human use [19]. In fact, curcumin is widely used worldwide for both food and pharmaceutical purposes showing important antioxidants, anti-inflammatory and anti-diabetic effects [18, 20–22]. In addition, curcumin showed cytotoxic effects in cancer cells demonstrating good effects and as an anticancer agent by inhibiting cell proliferation and inducing apoptosis in several cancer types [23–30].

Prostatic adenocarcinoma 22Rv1 cell line is a prostate cancer cell line derived from a human carcinoma xenograft (CWR22R) serially propagated in nude mice after the castration-induced regression and relapse of the parental androgen-dependent CWR22 xenograft [31, 32]. 22Rv1 cells are Prostate Specific Antigen (PSA)-positive, present mutations in p53 gene [33] and, although the androgen receptor (AR) is mutated at codon 874 (His to Tyr) close to the steroid binding pocket, the binding affinity of natural steroids to AR of 22RV1 cells is very similar to wild type AR demonstrating that a preservation of steroid specificity [34].

22Rv1 cells derive from an androgen-dependent primary prostatic carcinoma (Gleason 9) (CWR22) from a patient with osseous metastases, after repeated tumor regression and relapsed under castrated condition. These cells are androgen-sensitive but androgen-independent and are characterized by a low invasiveness [32, 35]. Thus, the 22Rv1 cell line is a useful tool to study castration-resistant prostate cancer (CRPC).

Since high curcumin concentrations (as well as other natural compounds) may exert cytotoxic effects also in normal cells, the use of the lowest concentration with anticancer effect is necessary to avoid/reduce normal cell damages. The studies reported in literature treat 22Rv1 cell line with doses of curcumin ≥ 10 µM and show a reduced cell proliferation, invasion and motility, increased apoptosis, and altered cell cycle [36–40]. None of the studies reported in literature investigated the possible effect of curcumin inducing cell senescence in this cell line.

To the best of our knowledge, there is only one study investigating the effect of low dose curcumin (5 µM) treatment in 22Rv1 prostate cancer cells. However, this study just showed that 5 µM curcumin treatment inhibited 22Rv1 cells growth [36]. However, this study did not evaluate if curcumin treatment at this concentration could alter cell cycle or induce cell senescence. Thus, the mechanism of action by which such a low dose curcumin reduces 22Rv1 cells proliferation is still unknown. The aim of this study was to find the best (ideally the lowest) curcumin concentration able to reduce 22Rv1 cell proliferation for potential enhancement of standard therapies in patients with CRPC. In addition, we were aimed to identify the mechanism by which curcumin obtain this effect.

## Materials and methods

### Cell culture and treatments

Androgen-independent 22Rv1 cell lines (ATCC/LGC Standards, Manassas, VA, USA) were grown in RPMI 1640 (Life technologies, Carlsbad, CA, USA) supplemented with 10% Fetal Bovine Serum (FBS; Gibco, Thermo Fisher Scientific, Waltham, MA, USA) and 100 U/mL penicillin and streptomycin (Gibco) at 37° C, 95% humidity and 5% CO_2_. The medium was changed every 2 to 3 days and cells were split 1:4 every 4 days. Cells were treated with 5, 15 and 30 µM curcumin (Merck, Darmstadt, Germany) for 24 h. Cell morphology was captured by using Nikon Eclipse Ti inverted light microscope fitted with a Nikon DS-L2 camera control.

### Flow cytometry analysis

Flow cytometry analysis was performed by Facs Canto II (Becton-Dickinson, Frank-lin Lakes, NJ) and computer software (BD FACSDiva, BD Biosciences). 7-Amino actinomycin D (7-AAD, BD Biosciences) cell viability flow cytometry protocol was used to detect non-viable cells. 7-AAD is a membrane impermeant dye that is generally excluded from viable cells. 7-AAD intercalates between cytosine and guanine bases of the DNA and allow discrimination of viable (7-AAD negative) from death cells (7-AAD positive). The cells were incubated 15 min at room temperature in the dark with 7-AAD and the fluorescence was detected in the far-red range of the spectrum (650 nm long-pass filter) and almost 100,000 cells were acquired for each sample.

Propidium iodide (PI) solution (MACS, Miltenyi Bio-tec) was used for cell cycle analysis as described below: 1 × 10^6^ cells were fixed cells in cold 70% ethanol overnight (+ 2 to + 8 °C), washed twice in cold PBS (+ 2 to + 8 °C), centrifuged at 2000 rpm for 10 min and discarded supernatant. Then, cells were incubated for 30 min at room temperature with propidium iodide at a final concentration of 1 µg/mL added by 1 U of Rnase, Dnase-free. Cells were analyzed immediately after the second incubation, without washing.

The PI intercalates into the major groove of double-stranded DNA producing a highly fluorescent signal when excited at 488 nm with a broad emission centered in the PE channel around 600 nm. Cells preparing for division will contain increasing amounts of DNA and display proportionally increased fluorescence. Differences in fluorescence intensity are used to determine the percentage of cells in each phase of the cell cycle.

### Clonogenic assay

Cells were plated at 500 cells/well in 6-well plates. The day after, the culture medium was changed with new medium with or without curcumin (5, 15 and 30 µM). Cells were kept in culture for 10 days until colonies were visible. Colonies were fixed for 20 min at room temperature in 4% paraformaldehyde and stained with 5% (w/v) crystal violet for 10 min at room temperature. Finally, the colonies were counted by using Nikon Eclipse Ti inverted light microscope fitted with a Nikon DS-L2 camera control.

### Western blot analysis

Cells were lysed by using the following lysis buffer: 0.1 M PBS, 0.1% (w/v) SDS, 1% (w/w) NONIDET-P40, 1 Mm (w/v) Na orthovanadate, 1 Mm(w/w) PMSF (phenyl methyl sulfonyl fluoride), 12 Mm(w/v) Nadeoxycholate, 1.7 µg/Ml Aprotinin, Ph 7.5. Cell lysates were centrifuged at 20,000 x g for 20 min at 4° C and the supernatants were aliquoted and stored at– 80 °C until use. Bradford protein assay (Bio-Rad Laboratories, Milan, Italy) was used to determine proteins’ concentrations. Protein samples were fractionated on 10% SDS-polyacrylamide gels (SDS-PAGE) and electrophoretically transferred (Trans-Blot^®^ Turbo™ Transfer System; Bio-Rad Laboratories Inc, Richmond, CA, USA) to nitrocellulose membranes. Non-specific protein binding was blocked with EveryBlot Blocking Buffer (Bio-Rad Laboratories) for 5 min at room temperature. Blots were incubated with the primary antibodies listed in Table 1 overnight at 4 °C. After washing with tris-buffered saline TBS/0.05% Tween 20 (TBS-T), blots were incubated with the appropriate secondary antibody conjugated with horseradish peroxidase (Amersham Italia s.r.l., Milano, Italy) diluted 1:5000 in TBS-T. Detection of bound antibodies was performed with the Clarity Western ECL Substrate (Bio-Rad Laboratories) and images were acquired with Chemidoc (Bio-Rad Laboratories). Bands were analyzed using the ImageJ software (https://imagej.nih.gov/ij/download.html) for quantification, and normalization was completed using β-Actin band intensities.

**Table 1:** List of antibodies used in this study

Antibody	Dilution	Company
Rabbit anti-Caspase-3 (#9662)	1:1000	Cell Signaling Technology, Danvers, USA
Rabbit anti-Cleaved- Caspase-3 (#9662)	1:1000	Cell Signaling Technology, Danvers, USA
Rabbit anti-p21 (#2947)	1:1000	Cell Signaling Technology, Danvers, USA
Mouse anti-p16 (sc-377412)	1:200	Santa Cruz Biotechnology, Inc. Dallas, US
Mouse anti-β-Actin (#sc-47778)	1:250	Santa Cruz Biotechnology, Inc. Dallas, US

### Statistical analysis

Statistical analyses were performed with Graph-Pad Prism (ver. 8) software. Data are reported as mean ± standard error of the mean (SEM). Pairwise comparisons were analyzed using Student’s t-test while comparisons between multiple conditions were done by one-way ANOVA. *p* < 0.05 was considered statistically significant.

## Results

### Cell apoptosis depends on curcumin concentrations

**Fig. 2:**
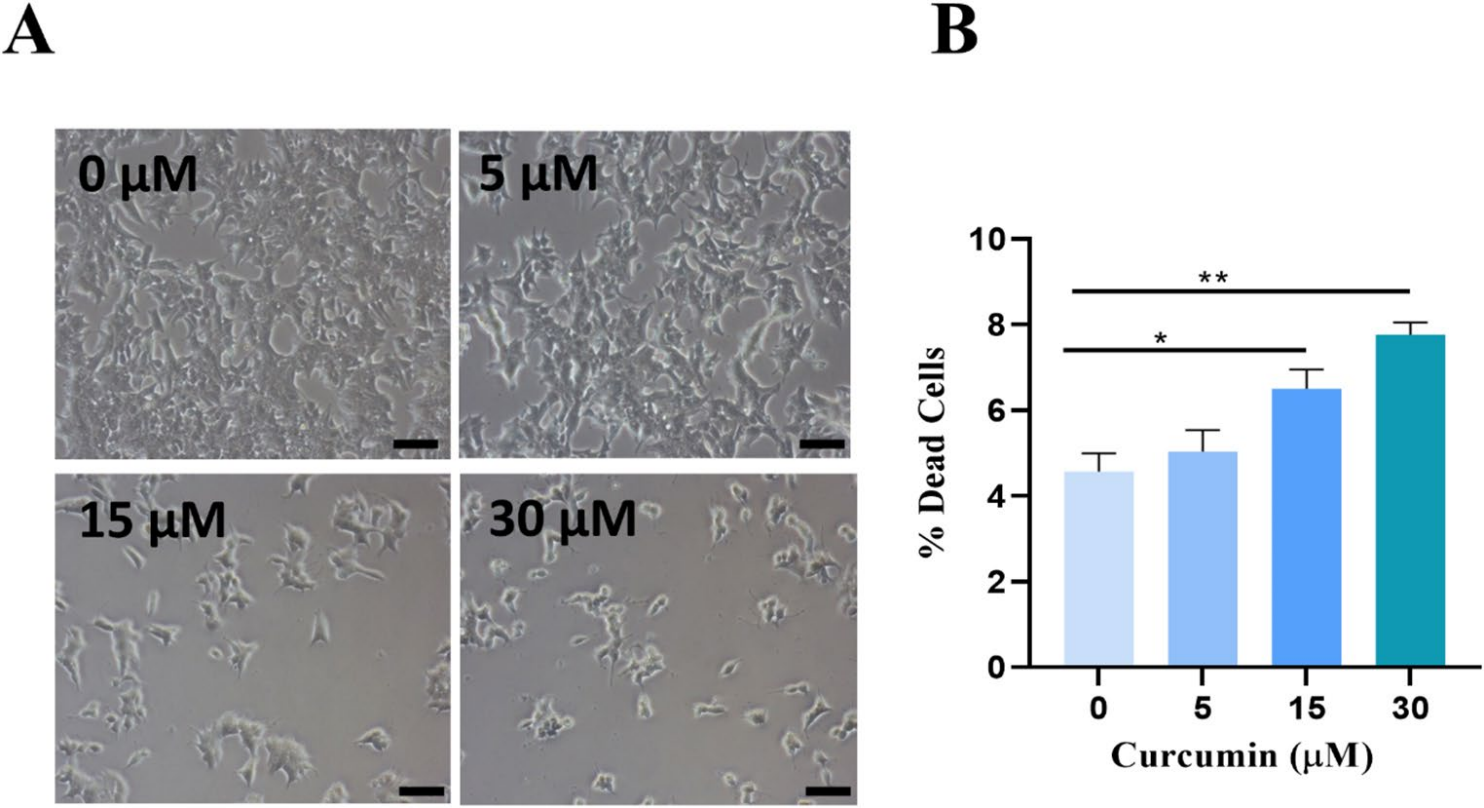
Curcumin effects on 22Rv1 cell morphology and cell death. (**A**) Morphology of 22Rv1 cells exposed to 5, 15 and 30 µM curcumin for 24 h. (**B**) Flow cytometric analysis of 7-AAD staining reporting the percentage of dead cells. Data are represented as mean ± SEM. *p < 0.05, **p < 0.01. Scale bars = 50 μm. Magnification: 10X

As shown in Figs. 2A, 22Rv1 cells exposed to increasing concentrations of curcumin (5, 15 and 30 µM) showed a lower confluence compared to the untreated cells which show the classic aspect of semi-confluent cultures with normal morphology. Moreover, the confluence of treated cells decreased in a concentration-depending manner. Morphological changes were less evident in cells treated with 5 µM of curcumin.

7-Aminoactinomycin D (7-AAD) staining is suitable for quantification of apoptosis in flow cytometry, this assay showed a statistically significant increase in cell death after treatment with 15 and 30 µM of curcumin compared with control. Treatment with curcumin 5 µM did not show a statistically significant increase in apoptosis (Fig. 2B).

**Fig. 3:**
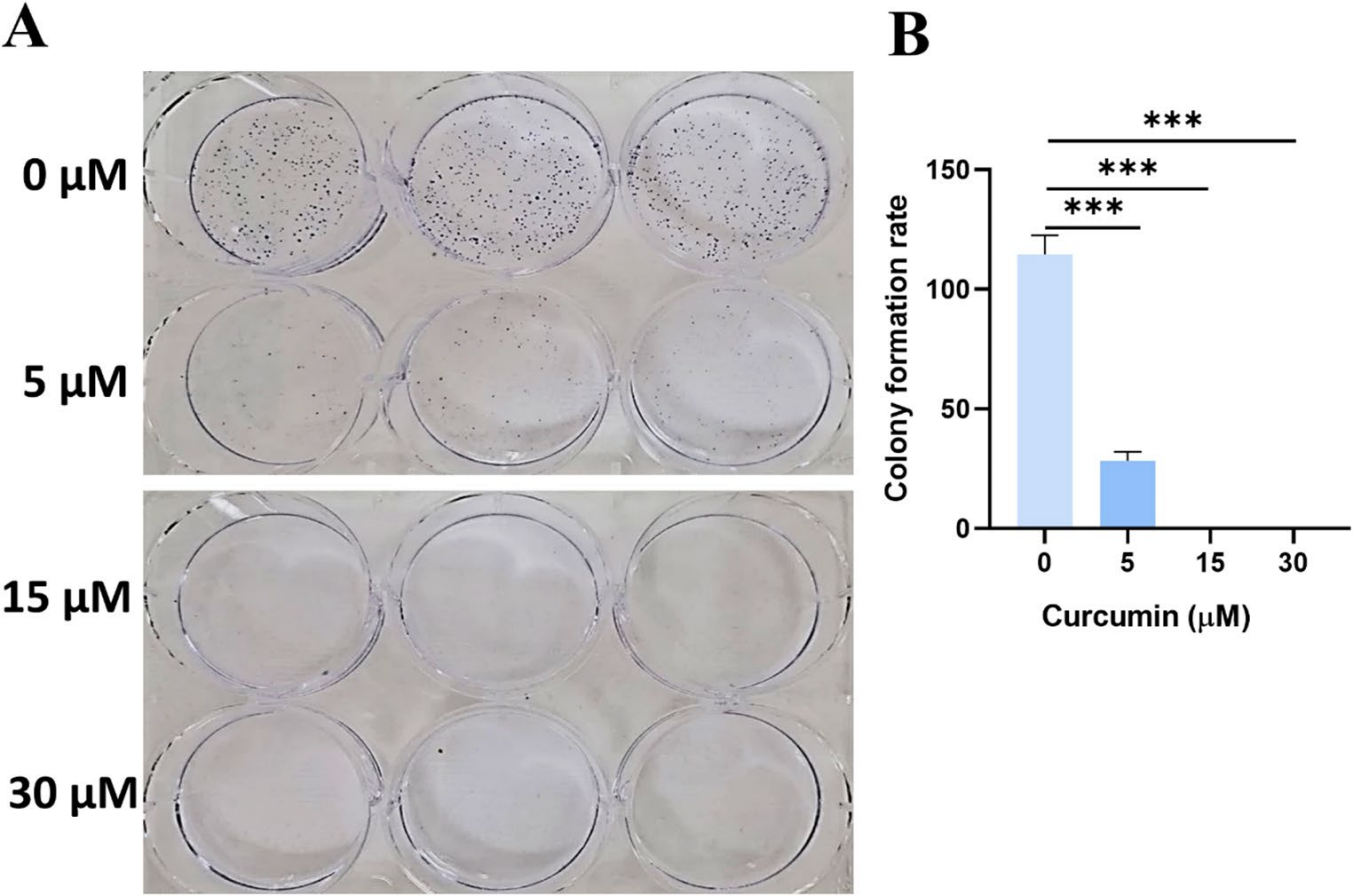
Effect of curcumin on 22Rv1 colony formation. All curcumin concentrations used showed a reduction in colonies’ number when compared with untreated wells. Graph shows mean ± SEM, ***P < 0.001

We performed a colony formation assay on 22Rv1 cells treated with increasing concentrations of curcumin (5, 15 and 30 µM) to confirm the anti-proliferative effect of curcumin (Fig. 3). Interestingly, we found a statistically significant decrease of colonies in all concentration of curcumin, but we noted that only the treatment of cells with 5 µM of curcumin showed vital cells. These data demonstrated clear antiproliferative effects of curcumin on 22Rv1 cells at all three concentrations used (5, 15 and 30 µM).

**Fig. 4:**
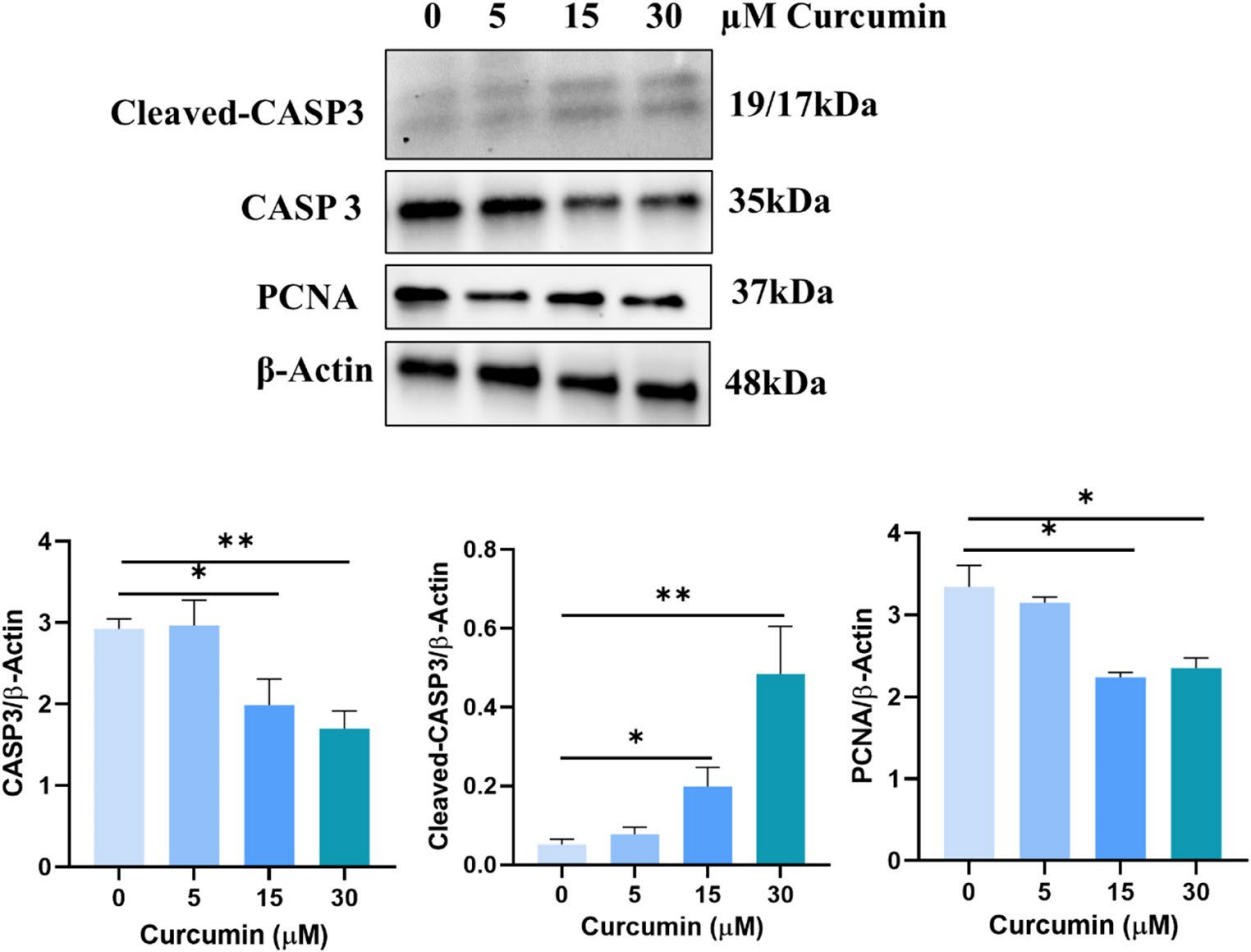
Effects of curcumin on caspase 3 activation and PCNA expression. 15 and 30 µM curcumin increased the activation of caspase 3 increasing the expression of its cleaved form. PCNA expression was decreased in15 and 30 µM curcumin treatments. Data are represented as mean ± SEM. *p < 0.05, **p < 0.01

These data were confirmed by western blotting analysis which showed a decrease in total caspase 3 expression due to an increase of the cleaved (active) caspase 3 expression only in cells treated with 15 and 30 µM curcumin. These curcumin concentrations also decreased PCNA expression. No statistically significant changes in total and cleaved caspase 3 and PCNA expression were found in cells treated with 5 µM curcumin (Fig. 4).

### Curcumin treatments promoted cell cycle arrest

**Fig. 5:**
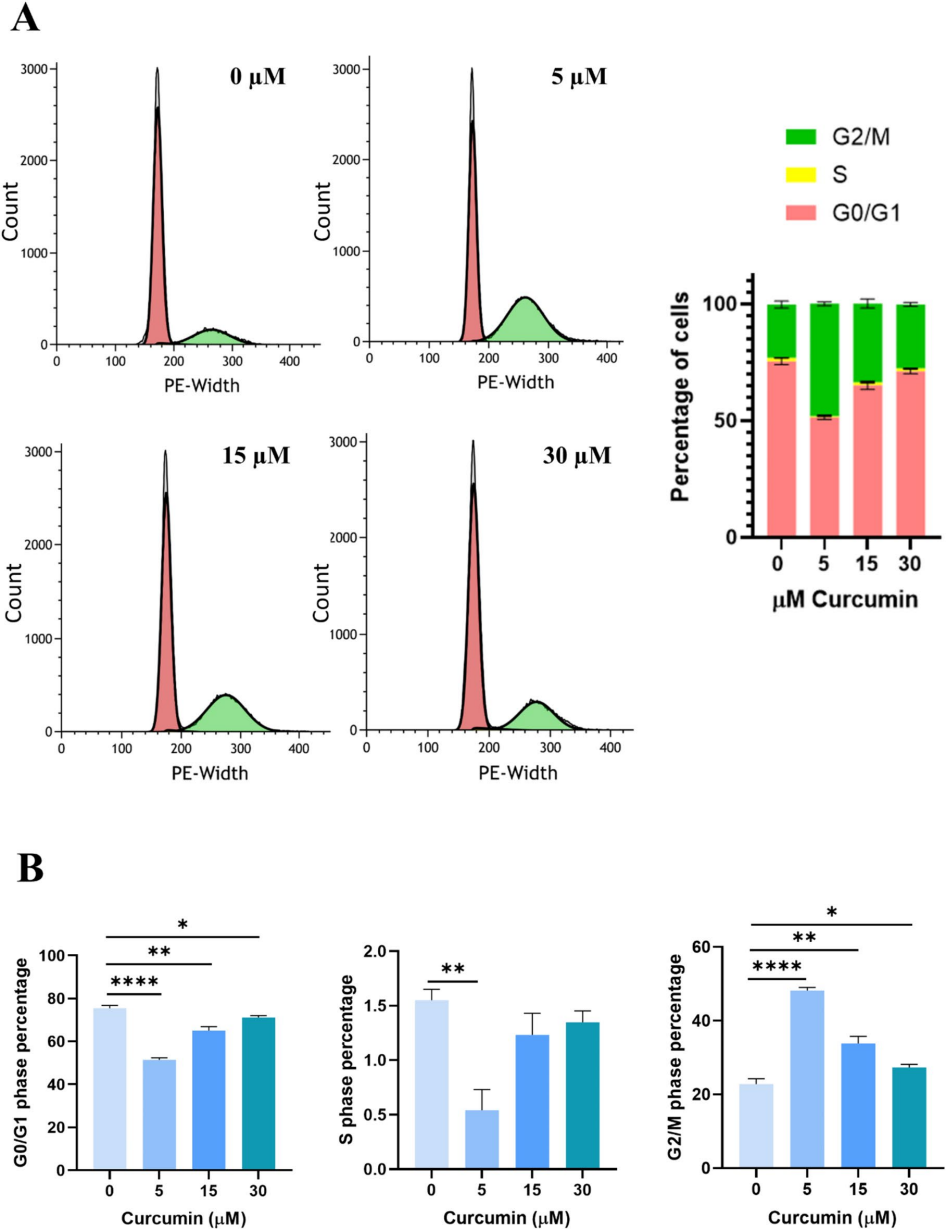
Effect of curcumin on cell cycle. **A**) Representation of cell cycle phases of 22Rv1 cells treated with 5, 15 and 30 µM curcumin. **B**) Representation of the single phase of cell cycle (G0/G1, S and G2/M). Data are represented as mean ± SEM. *p < 0.05, **p < 0.01, ****p < 0.0001

To evaluate the possible role of curcumin in altering cell cycle, we performed propidium iodide (PI) staining and evaluated the cells by flow cytometry analysis. As shown in Fig. 5A-B, all curcumin concentrations used decreased cell number in G0/G1 phase while increased the number of the G2/M phase compared to untreated cells demonstrating that curcumin induced a block in G2/M phase of cell cycle. In addition, cells number in S phase decreased only in 22Rv1 treated with 5 µM curcumin compared to untreated cells suggesting that this concentration differently modified the cell cycle compared to the higher concentrations.

### Cell death depend on curcumin concentrations

**Fig. 6:**
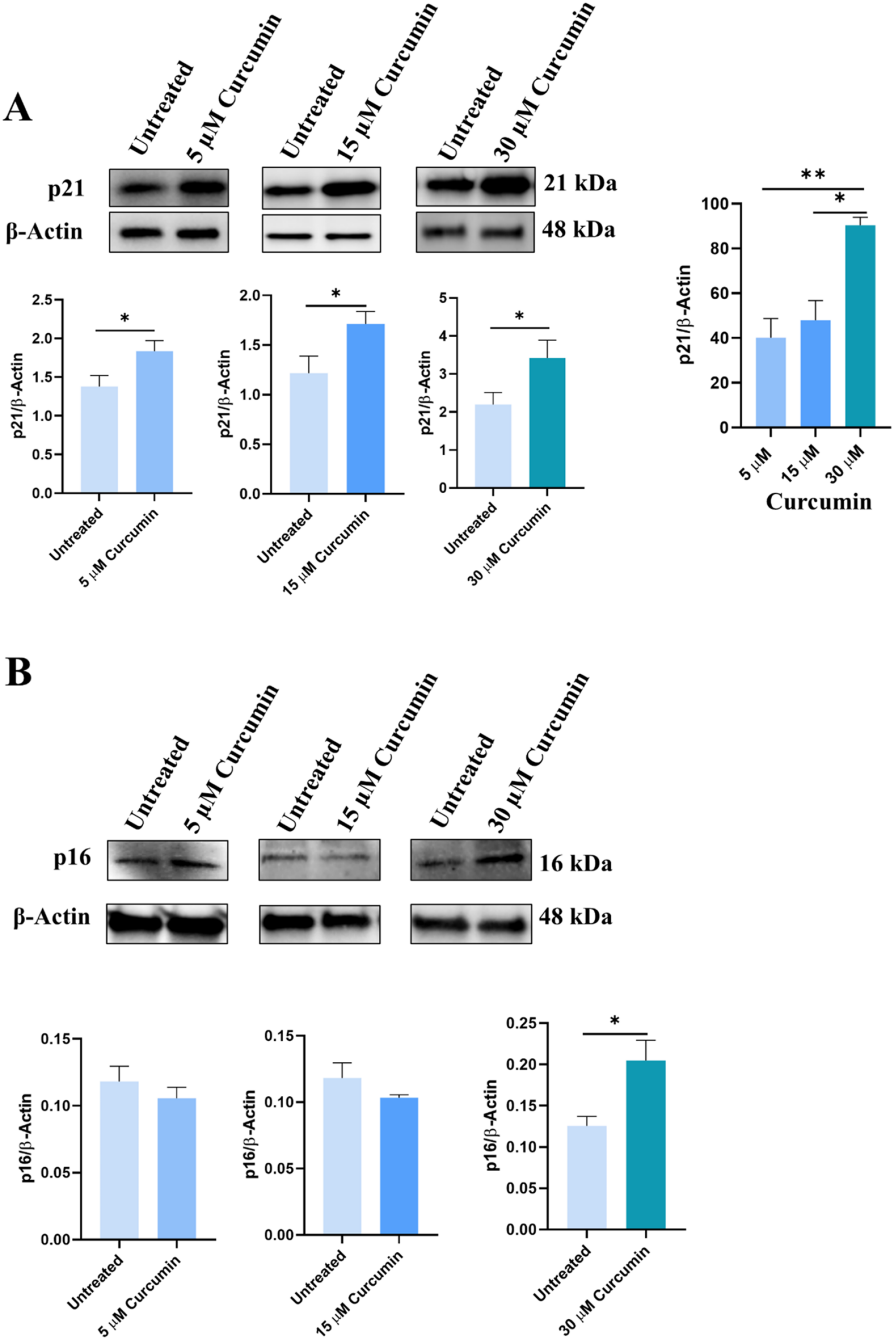
Effect of curcumin on p21 (**A**) and p16 (**B**) expression in 22Rv1 cells treated with 5, 15 and 30 µM curcumin. **A**) The last diagram on the right shows the differences in p21 expression in relation to the different concentrations of curcumin. **B**) diagrams show p16 levels at different concentrations of curcumin. Data are represented as mean ± SEM. *p < 0.05, **p < 0.01

Since 5 µM curcumin did not induce apoptosis, differently to 15 and 30 µM concentrations (see Fig. 4), but induced G2/M phase block as the other curcumin concentrations (see Fig. 5), we hypothesized that curcumin may induce cell senescence. To this aim, we performed a Western blotting assay to assess p21 and p16 expression, two known markers of cell senescence [41]. As shown in Fig. 6A, p21 protein expression was significantly increased in all curcumin concentrations used compared to the untreated cells. In addition, the increase of p21 was dependent on the increase of curcumin concentration since p21 expression was significantly increased at 15 and 30 µM of curcumin compared to 5 µM of curcumin treatment. However, as shown in Fig. 6B, p16 protein expression significantly increased only at 30 µM curcumin concentration.

## Discussion

The development of resistance to drugs such as enzalutamide and abiraterone for PCa treatment is still a major challenge. In addition, elderly need to tailored approaches to PCa treatment because of more medical comorbidities and less physical functionality and frailty to ensure better tolerance and a good outcome of treatments. For instance, frailty is exacerbated by ADT since this therapy depletes testosterone whose decrease is the main cause of frailty [15]. In fact, chemotherapy drugs and even radiotherapy could cause serious adverse reactions so the use of natural compounds alone or in association with drugs have the advantage of lower side effects [42]. Natural compounds are often described as therapeutic agents with low side effects. This characteristic is of great interest, especially in the treatment of elderly patients which often have comorbidities. Different studies analyzed the effect of curcumin in suppressing cell proliferation and invasion in different tumors including prostate cancer [39, 43]. Plant-based natural products are the principal sources of chemotherapeutic drugs but often the anti-tumoral activity of different compounds causes cytotoxic effects in tumoral but also in normal tissues [44, 45]. Thus, the concentration of these compounds (also natural) should be kept as low as possible. In this study, we highlighted the fact that low doses of curcumin (5 µM) show important anticancer effects in prostate cancer cell line 22rv1. Curcumin is known to have a cytotoxic effect by inducing apoptosis through the cytochrome c translocation from the mitochondria to the cytosol promoting the subsequent activation of caspase 3 [46]. Our results demonstrated that 15 or 30 µM curcumin promoted caspase activation enhancing cleaved caspase 3 expression in 22Rv1 cells. However, 5 µM curcumin did not affect cell apoptosis. Moreover, all curcumin concentrations tested in this study showed that curcumin can block 22Rv1 cells in G2/M phase and inhibit cell proliferation. Moreover, 5 µM curcumin was able to reduce the number of cells in the S phase. Thus, curcumin can modulate 22Rv1 cell cycle blocking cells in G2/M phase, a critical mechanism for the inhibition of cancer cell proliferation that can contribute to the suppression of tumor growth and progression. Our data concerning senescent phenotype supports previous studies performed in other cancer cells [47, 48]. Senescence is strongly correlated with the accumulation of p21 and p16 since they play a key role in cell cycle arrest [41] but we observed a variable amounts of these proteins in cells treated with 5, 15 and 30 µM curcumin. In fact, we showed an enhanced expression of p21 in 22Rv1cells in every experimental condition, but the p21 expression was increased with increasing of curcumin concentration. However, p16 levels were significantly increased only in cells treated with 30 µM curcumin. These results can be explained by the fact that p21 is prominently expressed at early stages of senescence process, whereas p16 appears only later [49, 50]. Then, 30 µM curcumin treatment may induce cell senescence earlier than the 5 and 15 µM curcumin treatment since all treatments were performed for 24 h.

These data confirmed that these cells acquired an increasingly senescent phenotype as the dose of curcumin increase. Cell senescence is characterized by a permanent cell cycle arrest without apoptosis and can be induced by oncogenes, chemotherapy, radiotherapy, cytokines and oxidative stress. Senescence plays a key role in cancer therapy since it promotes tumor suppression [51]. To this point, we made two hypotheses: (1) low dose curcumin could be used in combination with ADT or radiotherapy to improve treatment efficiency by blocking cell proliferation; (2) since low dose curcumin (5 µM) does not cause cell death, this treatment may not be clinically efficient, and higher doses may be necessary. The combination of ADT + 5 µM of curcumin could not sustain the non-proliferative state of the cells, indicating that this double treatment could not be curative because the cell proliferation is decreased but not inhibited. Otherwise, the double treatment should be carried out with higher doses of curcumin for example with 15 µM that ensures cell death. In fact, cell senescence (demonstrated by the high levels of p21 protein expression) is followed by apoptosis (demonstrated by increased cleaved caspase 3 expression) in cells treated with 15 and 30 µM curcumin while this does not happen at with 5 µM curcumin treatment, which only maintain the senescent status but not induces apoptosis (increased p21 expression but no alteration in cleaved caspase 3 expression). However, 30 µM curcumin induced cell senescence earlier than the 5 and 15 µM curcumin treatment (increased p16 levels only at 30 µM curcumin).

The combination of radiotherapy + 5 µM of curcumin could be the key to keeping a portion of senescent cancer cells that can be attacked by radiotherapy. Recently, anticancer activity of oral curcumin in patients with prostate cancer was evaluated [52]. A randomized double-blind, placebo-controlled trial was performed on patients with prostate cancer who received intermittent androgen deprivation (IAD). This study reported that the oral curcumin lowered PSA levels, but it did not modify the clinical outcomes. These authors tested a unique dose of 240 mg of curcuminoid powder in capsule form in combination with IAD. Another study on curcumin in combination with docetaxel and prednisone in patients with CPRP was performed [53]. Unfortunately, this study did not involve control groups. These studies demonstrated that curcumin could have a potential role in prostate cancer care.

## Conclusion

In conclusion, our data demonstrated the cytotoxic effect of curcumin at higher concentration while at lower concentration showed an important cytostatic effect by inducing cell senescence. Thus, we suggest that curcumin concentrations and its use in combination with the appropriate therapy could bring valuable clinical improvements.

## Data Availability

No datasets were generated or analysed during the current study.
